# Genetic Instability in Cyanobacteria – An Elephant in the Room?

**DOI:** 10.3389/fbioe.2014.00012

**Published:** 2014-05-02

**Authors:** Patrik R. Jones

**Affiliations:** ^1^Department of Life Sciences, Imperial College London, London, UK

**Keywords:** cyanobacteria, genetic instability, metabolic engineering, biotechnology, mutation, recA

## Abstract

Many research groups are interested in engineering the metabolism of cyanobacteria with the objective to convert solar energy, CO_2_, and water (perhaps also N_2_) into commercially valuable products. Toward this objective, many challenges stand in the way before sustainable production can be realized. One of these challenges, potentially, is genetic instability. Although only a handful of reports of this phenomenon are available in the scientific literature, it does appear to be a real issue that so far has not been studied much in cyanobacteria. With this brief perspective, I wish to raise the awareness of this potential issue and hope to inspire future studies on the topic as I believe it will make an important contribution to enabling sustainable large-scale biotechnology in the future using aquatic photobiological microorganisms.

In our laboratory, we are like many others interested in engineering the metabolism of cyanobacteria with the objective to convert solar energy, CO_2_, and water (perhaps also N_2_) into end-products of commercial value. In comparison to *Escherichia coli*, engineering cyanobacteria can be a slow experience. Imagine if after several rounds of painfully slow construction, transformation, selection, and verification, that one of the modifications is undone. Or, consider crafting an ideal biotechnological strain that is cultivated at a large scale and finding out mid-way through a commercial run that productivity suddenly and unexpectedly drops. It may be caused by one or few nucleotide mutations or a complete loss of a varyingly sized fragment, both resulting in either a loss of gene expression or the functionality of the RNA or protein it encodes. For the purpose of this perspective, we refer to these unwanted changes as “genetic instability” and ignore other potential reasons for loss of productivity such as contamination with other biological species that outcompete or even consume the biotechnological catalyst (Wang et al., 2013).

Our unpublished observations and personal communication with colleagues suggest that such instances of genetic instability are commonly observed. Chances are it will not be reported, nor will the nature of the “instability” be investigated. That is often the fate of negative results; few people are willing to spend resources in chasing up loose ends, which are unlikely to enhance or result in a publication. Instead, they will try it again or try something different, hoping it will work the next time. If such genetic instability is indeed common, yet seldom reported, it is worthy of being called an elephant in the room, or at least a baby elephant; important, obvious, yet largely ignored.

## Adaptive Evolution as a Response to Environmental Stress

Cyanobacteria have evolved over a long period of time to be successful in their native dynamic environments. Following the “capture” of some of these strains from their natural environment and maintenance under artificial conditions that are likely to be different (i.e., laboratories), it can be asked whether such strains have evolved as a response to this change in their environment. An answer to this may come from examining articles where cyanobacteria have been re-sequenced or sub-strains originating from a single ancestor have been sequenced (Kanesaki et al., 2012; Trautmann et al., 2012). The history of cultivation and exposure to stress is likely to remain unknown in these cases, although it can be assumed that routine laboratory maintenance presents an environment that differs from that in nature (Ikeuchi and Tabata, 2001).

Four sub-strains of *Synechocystis* sp. PCC 6803 (GT-Kazusa, PCC-N, PCC-P, and PCC-M) that originated from the same original isolate [Isolated in 1968; GT-Kazusa was first sequenced in 1996 (Kaneko et al., 1996)] have been maintained in different laboratories. When all four strains recently were (re-)sequenced (Kanesaki et al., 2012; Trautmann et al., 2012), genetic differences were indeed identified. The changes [45 in total, mainly single nucleotide polymorphism (SNPs, i.e., exchange of nucleotides), six 1 bp deletions or insertions, four larger deletions (12–154 bp) and the remainder mobile elements] are visually mapped out in Figure 3 in the work of Trautmann et al. (2012). The impact of those genetic changes on functionality are difficult to connect without further study except where concrete changes in the phenotype (e.g., phototaxis, glucose-uptake, and clumping) have been observed in strains where the amino acid sequence of known proteins have been modified. Interestingly, some of the genes that were affected are known to be regulators associated with stress responses, including *pmgA* (Takahashi et al., 2008), *hik10* (Shoumskaya et al., 2005), *hik25*, and *hik33* (Mikami et al., 2002).

## Genetic “Instability” in Cyanobacteria in Response to Metabolic Engineering

In *E. coli*, metabolic engineering has under some conditions been shown to result in genetic instability that at least in part correlates with the level of protein expression and also presence of repeat elements (Tyo et al., 2009; Sleight and Sauro, 2013). Like with *E. coli*, cyanobacterial strains are in many cases engineered successfully and instability is not observed, e.g., ethanol (Dienst et al., 2014) and isoprene (Bentley et al., 2014). However, a handful of reports of genetic instability in engineered cyanobacteria exist. Takahama et al. (2003) introduced an expression-construct for an enzyme called ethylene forming enzyme (*efe*) into *Synechococcus elongatus* PCC 7942. Although strains that survived the selective pressure were able to synthesize ethylene, they also exhibited a yellow–green appearance. Healthy green–blue variants soon appeared from colonies streaked out from the original transformed culture; however, such strains contained a truncated *efe* gene and did not produce ethylene. The phenomenon was reproducible as serial dilution consistently resulted in the transformation of a population of “unhealthy” ethylene-producers into a population of predominantly healthy strains incapable of ethylene formation. Also the mechanism of disruption was reproducible, with repetitive stretches in the *efe* gene disrupted in each examined case. Altogether, this suggested that expression of the *efe* gene product had a negative effect on the growth of *S. elongatus* PCC 7942 and that repetitive gene hot-spots were modified with a higher frequency than average. We were curious to understand this further and constructed a plasmid expression system for *efe* in *Synechocystis* sp. PCC 6803 with the hope of enabling a model system for genetic instability in cyanobacteria. In contrast to what was observed in *S. elongatus* PCC 7942, however, we observed no sign of instability in *Synechocystis* sp. PCC 6803 even with strains maintained as liquid cultures for more than 6 months (Guerrero et al., 2012).

Other examples also exist. Jacobsen and Frigaard (2014) constructed a mannitol-producing *Synechococcus* sp. PCC 7002 strain in which one particular construct variation consistently exhibited a loss of productivity and an inability to achieve complete segregation. Further analysis revealed a single base frame-shift resulting in the loss of mannitol productivity, and restoration of growth, reminiscent of the observations with *efe* (Takahama et al., 2003). Similarly, Angermayr et al. (2012) found that one slowly growing *Synechocystis* sp. PCC 6803 strain, that was engineered to produce lactic acid, appeared to revert to the wild-type growth rate. Analysis of one of the introduced parts (*sth*, which encodes a soluble NADPH:NADH transhydrogenase) in strains with improved growth revealed a duplication event causing the formation of premature stop codons. In another example, a single nucleotide mutation in *atoB*, one of several genes introduced to *Synechococcus* sp. PCC 7942 in order to produce isopropanol, was repeatedly found. This caused a change in one amino acid residue, which resulted in a reduction but not elimination of enzyme (and pathway) functionality (Kusakabe et al., 2013). Genetic instability was also observed in an earlier study where an electron-transfer protein (PetE) was over-expressed in the same species (Geerts et al., 1995). After the introduction of a second antibiotic selection marker in a strategic position, however, stable expression was observed following selection with the new antibiotic. Subsequently, the construct was stably maintained for over a year even in the absence of antibiotics, suggesting that the construct only was unstable up until the point at which complete segregation had been achieved.

## RecA

The observations of genetic instability in cyanobacteria mentioned above seem to relate to structural (i.e., changes in genetic sequence) rather than segregational instability (characterized by loss of entire modifications, typically observed with plasmids). In *E. coli*, these biotechnologically deleterious incidents have been found to be attenuated by the deletion of *recA* (Tyo et al., 2009), encoding a key enzyme for DNA recombination. In early studies, however, the complete elimination of the closest homolog to *E. coli* RecA (sll0569, 77% amino acid similarity in *Synechocystis* sp. PCC 6803; see also Table 1) in cyanobacteria was not possible despite repeated attempts, suggesting that RecA is very important in photosynthetic prokaryotes (Murphy et al., 1990). Minda et al. (2005), however, were subsequently able to obtain a fully segregated deletion mutant of *recA* in *Synechocystis* sp. PCC 6803 by carrying out the selection procedure under initially dark and thereafter low-light conditions. The *recA* mutant was highly sensitive to UV-light but displayed a similar rate of growth as the wild-type strain under low-light conditions. They concluded that RecA was essential under conditions where DNA was damaged and that high light in earlier studies had prevented the complete elimination of *recA* as a result of such damage. As it is likely challenging to fully control the quantity of light emanating from sunshine under outdoor large-scale conditions, the deletion of *recA* may therefore not be applicable to aquatic photosynthetic biotechnology in order to enhance genetic stability.

**Table 1:** **A selection of genes associated with both the production and avoidance of genetic mutations in *E. coli* and their homologs in cyanobacteria**.

*E. coli*	6803	7942	7002	7120

**METHYL-DIRECTED DNA REPAIR BY mutHLS COMPLEX**				
mutL	slr1199	synpcc7942_1780	SYNPCC7002_A0649	alr3055
mutS	sll1165	synpcc7942_2247	SYNPCC7002_A0178	alr3638
mutH	−	−	−	−
**RECOMBINATIONAL REPAIR**				
recA	sll0569	synpcc7942_0348	SYNPCC7002_A0426	all3272
recJ	sll1354	synpcc7942_1886	SYNPCC7002_A0980	alr5127
recF	sll1277	−	SYNPCC7002_A2277	all3374
**SOS RESPONSE**				
LexA	sll1626	Synpcc7942_1549	SYNPCC7002_A1849	alr4908
dinB (polIV)	−	−	−	alr7254
polB (polII)	−	−	−	−
umuCD (polV)	sll5122-23	synpcc7942_1549-50	−	−
uvrA	slr1844	Synpcc7942_0848	SYNPCC7002_A1468	alr3716
RuvA	sll0876	Synpcc7942_2298	SYNPCC7002_A1270	all0375
RpoS	sll0184 (sigC)	Synpcc7942_1557	SYNPCC7002_A0364	alr4249

*The presence or absence of a corresponding homologous *E. coli* gene in cyanobacteria was determined by BLASTP using KEGG with a probability cut-off with an *E*-value of 2e^−10^ and at least 50% amino acid sequence similarity as compared to the full-length translation product of the corresponding *E. coli* gene. The minus sign (−) indicates that a gene satisfying the above criteria was not found. 6803, *Synechocystis* sp. PCC 6803; 7942, *Synechococcus elongatus* PCC 7942; 7002, *Synechococcus* sp. PCC 7002; 7120, *Anabaena* sp. PCC 7120*.

Several other genes with a role in both DNA repair and mutation have also been identified in *E. coli* (Table 1). The complement of genes in this small selection is not fully represented in model cyanobacteria species, particularly genes associated with the “*SOS*” response. It is of course possible that proteins with the corresponding functionality in *E. coli* exist in cyanobacteria, yet are not identified by simple BLAST.

## Is Genetic “Instability” Inducible in Cyanobacteria?

It is in *E. coli* at least (Fijalkowska et al., 1997; McKenzie et al., 2000). The SOS response is an induced mechanism for both rapid repair of DNA (Kuzminov, 1999) and adaptive evolution (McKenzie et al., 2000). The latter is characterized by the promotion of mutations, rather than prevention, in order to achieve offspring that have adapted to the new growth-limiting conditions (McKenzie et al., 2000) and therefore may survive better. Examples of these conditional changes include the switch from an assimilable to a non-assimilable (by the wild-type) substrate or exposure to antibiotic agents (Michel, 2005). The mutator activity associated with the adaptive SOS response related mutations requires constitutive RecA activity that is induced by stalling DNA replication or binding of RecA to single-stranded DNA (McKenzie et al., 2000), though the mutation does not necessarily occur exclusively at the site of the stall (Fijalkowska et al., 1997). The link between the induction of a general SOS response and changes in environmental or physiological growth conditions, the original reason for adaptive evolution, however, is less clear.

LexA is the first main control element for the induced mutagenic SOS response in *E. coli*. Although a homolog of *lexA* exists in cyanobacteria (Table 1), it does not appear to have a role in DNA repair, at least in *Synechocystis* sp. PCC 6803 (Patterson-Fortin et al., 2006). As the LexA-regulon is essential for induced mutation in *E. coli*, it would be interesting to understand how this mechanism is managed in cyanobacteria, and whether it is realized or not, in the absence of a DNA repair associated LexA. Interestingly, a computational analysis across a large number of cyanobacterial genome sequences suggests that LexA has a similar role to that in *E. coli* in most cyanobacteria, though not in *Synechocystis* sp. PCC 6803 (Li et al., 2010), the most common model species used for cyanobacteria research.

The sigma factor RpoS (Sigma 38), a regulator of a generalized stress response (Battesti et al., 2011), is a second control element for DNA mutation in *E. coli*. Here, the role of SOS in RpoS-regulated DNA mutation is solely to upregulate the expression of DinB (Galhardo et al., 2009), an error-prone DNA polymerase, which does not have any close homologs in model cyanobacteria (Table 1). The closest homolog of RpoS in *Synechocystis* sp. PCC 6803 is SigC, though SigC displays slightly greater amino acid sequence similarity to *E. coli* RpoD (Sigma 70). As the two *E. coli* sigma factors, RpoS and RpoD, have considerably different roles, yet highly similar sequences, it is challenging to elucidate what role any homologs may have in other species.

In conclusion, the search for genes involved in the repair and/or mutation of DNA in cyanobacteria, that are homologous to those in *E. coli*, is not straightforward. It is possible that all components needed to implement effective mutation (or relax the quality of repair) of DNA under particular conditions may be present in cyanobacteria, only we do not yet know any or all components that constitute the required system. Clearly, some components will differ. For example, LexA has a completely different role in cyanobacteria than in *E. coli*, and there does not appear to be a trace of some of the error-prone DNA polymerases. The external environment under which cyanobacteria evolved is also likely to be markedly different from the environments that *E. coli* evolved in.

## Response of Genes with a Role in DNA Repair and Mutation to Stress

If DNA mutations are inducible, one possibility is that genes with a role in DNA repair and mutation (Table 1) respond to conditions in which there is environmental stress or a metabolic burden. In recent omics studies, the whole-system response of cyanobacteria has been evaluated with strains that have been exposed to potential fuel production targets (Liu et al., 2012; Qiao et al., 2012; Tian et al., 2013; Zhu et al., 2013). Notably, in all studied cases, the exposure to the solvent had a measurable negative effect on growth, and the relative quantity of several hundred transcripts and/or proteins was modified. Of those genes listed in Table 1 (genes associated with DNA repair or mutation), only two were affected by any of the treatments: The accumulation of UvrA (slr1844) was enhanced by ethanol exposure (Qiao et al., 2012) and the accumulation of RecF (sll1277) was enhanced by hexane exposure (Liu et al., 2012).

In contrast, Dienst et al. (2014) analyzed the transcriptomic response of *Synechocystis* sp. PCC 6803 to the introduction of an ethanol pathway. Surprisingly, even though the presence of the ethanol pathway resulted in a marked negative effect on growth, only three mRNAs displayed a clear quantitative difference, none of which is known to be associated with genetic modifications or stress. This result is surprising on two levels: Firstly, there was no reported loss of productivity (an indicator of genetic instability) over presumed repeat experiments, despite a marked negative effect on growth and cultivation for 18 days. Secondly, there was no immediate (i.e., not evolved) transcriptional response to either the pathway or reduced growth. This contrasts markedly to the above-mentioned studies where solvents (including ethanol) were added externally to the medium. Was the negative effect on growth in the Dienst study not sufficiently strong? The ethanol concentration that the engineered strains reached [0.6% (v/v)] was several-fold lower than in studies where it was added externally, and in previous studies, there was no measurable change in the growth rate at this concentration (Kämäräinen et al., 2012). The observed negative growth effect may therefore at least in part be caused by an effect of the metabolic engineering (e.g., antibiotic resistance, heterologous protein over-expression, metabolic interference, intermediate toxicity), rather than as a direct consequence of the continuously increasing concentration of ethanol. Still, if genetic instability only exploits differential fitness to allow mutations to propagate, the cause of poor fitness (by engineering or environmental manipulation) should not matter.

## Alternative Solutions

Most initial engineering tools in cyanobacteria did not allow for user-controlled regulated protein expression, and such non-silenceable systems were used in all the reports of genetic instability in cyanobacteria described above, with one exception (Geerts et al., 1995). Recently, there has been progress toward the development of regulatable promoters for engineering cyanobacteria that are capable of user-controlled regulation of protein expression over a wide dynamic range. Although a perfect system is arguably not yet in sight, several promising alternatives are at least available (Guerrero et al., 2012; Huang and Lindblad, 2013). Whether their use may limit genetic “instability” or not is an interesting question that warrants further investigation.

## Conclusion

The limited amount of available information makes it difficult to assess how great an issue genetic instability really is toward the aim of developing aquatic photobiological biotechnology. Several other issues also exist including excessively expensive cultivation infrastructure and culture crashes as a result of biological contamination/invasion. In the absence of a *recA*-based solution, how can we prevent genetic instability? Currently, there are no other solutions in the literature that are obvious. At first, however, it would be good to at least confirm on an analytical level that genetic instability is indeed an issue and if so to determine whether or not it is inducible in cyanobacteria. It would be advisable to extend such studies to more than one model cyanobacteria species as they appear to be quite different in this respect, see (Guerrero et al., 2012) and Table 1. Thereafter, the development of a model system that is not excessively unstable, yet also not too stable, would pave the way for both targeted and non-targeted screens for factors that are essential for the process. This may then lead on to possible solutions to minimize the negative impact of genetic instability in cyanobacteria for both fundamental and applied sciences, and thereby contribute toward the development of economically sustainable aquatic photo biotechnology using engineered biology in a hopefully not too distant future.

## Conflict of Interest Statement

The author declares that the research was conducted in the absence of any commercial or financial relationships that could be construed as a potential conflict of interest.
